# Implementation of a hospital-based end-of-life and bereavement care program in a latin American middle-income country. A source of light and compassion in the midst of cloudy times

**DOI:** 10.1186/s12904-024-01522-3

**Published:** 2024-07-29

**Authors:** Ximena Garcia-Quintero, Eddy Carolina Pedraza, María Isabel Cuervo-Suarez, Isabel Correa^, Justin N. Baker, Michael J McNeil

**Affiliations:** 1 Department of Global Medicine, St. Jude Children’s Research Hospital, 262 Danny Thomas Place, Mail Stop 260, Memphis, TN 38105 USA; 2 Faculty of Health Sciences and Medicine, University of Lucerne, Alpenquai 4, Lucerne, 6005 Switzerland; 3 Pediatric Palliative Care, Maternal and Child Department, Fundación Valle de Lili, Avenida Simon Bolivar, Cra. 98 # 18-49, Cali, 760032 Colombia; 4 Facultad de Ciencias de la Salud, Universidad Icesi, Cali, 760032 Colombia; 5 Division of Quality of Life and Pediatric Palliative Care, Stanford Medicine Children’s Health, Palo Alto, CA 94304 USA; 6 Division of Quality of Life and Palliative Care, St. Jude Children’s Research Hospital, Memphis, TN USA

**Keywords:** End of life, Bereavement care, Low-and middle-income countries, Children, Bereaved families, Pediatric palliative care

## Abstract

**Background:**

The death of a child is one of the most devastating events a family can face, resulting in significant physical and psychosocial morbidity. Bereavement support programs have been developed in high-income contexts to address this need. However, little is known about implementing bereavement programs in low-and middle-income countries (LMICs). Here, we describe the implementation of a bereavement program for parents whose children died due to cancer or other catastrophic illnesses.

**Methods:**

We conducted a retrospective analysis to describe the implementation of a hospital-based End of Life (EoL) care and bereavement program. This program was developed in several stages, including an assessment of bereaved families, development program guidelines, staff training, piloting of the program, refinement, and standardization. The program was developed between 2019 and 2021 in a nonprofit, teaching hospital and referral center for southwestern Colombia.

**Results:**

Several tools were developed as key components of the bereavement program: a virtual bereavement course; guidance for EoL and bereavement communication and care, memory making, and follow-up calls; a condolence letter template, and group support workshops. A total of 956 healthcare professionals were trained, 258 follow-up calls to bereaved parents were made, 150 individual psychological follow-ups to parents with complicated grief occurred, 79 condolence letters were sent, and 10 support group workshops were carried out. Challenges were identified and overcome, such as limited resources and staff, and cultural perceptions of death. In 2021, this program received an award by the hospital as the Best Strategy to Humanize Healthcare.

**Conclusions:**

This study highlights the feasibility of developing and implementing EoL and bereavement care programs for parents and families within hospitals in LMICs. Lack of resources, staff, and training are some of the identified challenges to implementation. Utilizing methodological tools allows us to identify facilitator factors and deliverable outcomes of our EoL and bereavement program. This model provides a valuable framework for resource-limited settings.

**Supplementary Information:**

The online version contains supplementary material available at 10.1186/s12904-024-01522-3.

## Background

The death of a child is one of the most devastating events parents can face [1–3]. Parents, unprepared for such a scenario, often liken it to a dark, wet blanket [4] or a hole in their hearts [5]. A bereaved parent may face a range of emotions described as dreadful pain, incomprehensible emptiness [5–7], sadness, uncertainty, fear [8], and guilt, which may impact their psychological [9] and physical health [10–13], as well as their ability to socially integrate with their community [14].

Bereavement care after the death of a child has been proposed as a standard of care to support parents and families [15–17]. Interventions such as funeral assistance, condolence and anniversary letters, remembrance meetings, telephone follow-up [18], support groups, psychological follow-up (if needed), and continuity of care prior to and following the death of a child are all potential elements of bereavement care programs [19]. Therefore, multidisciplinary teams and the transition from hospital to community care are essential tools in these programs [16, 20, 21]. A lack of knowledge, time, and resources for bereavement care have been proposed by physicians as some of the barriers to implementing support for families after the death of a child [22]. Additionally, most bereavement programs and interventions that have been described are from high-income countries (HICs) [18, 20, 23–26], with little known about the support of parents and families of children who die in low-and middle-income countries (LMICs) [27] where the proportion of childhood mortality is significantly higher [27–30].

Colombia is one such country. Although gains have been made to decrease infant and child mortality [31], rates are still higher than those of other upper-middle income countries [32]. However, to our knowledge, no bereavement support program has yet been standardized or published in Colombia or other countries within Latin America. With this gap in mind, our paper aims to describe the implementation of a hospital-based End of Life (EoL) care and bereavement program focused on parents and families after the death of a child (0-18years old) in Cali, Colombia. The overarching goal of the EoL and bereavement program was to provide a standardized guidelines and support for bereaved parents who have lost a child under 18 years old, including perinatal and pediatric deaths in the hospital, in a standardized and institutionalized manner.

## Methods

A descriptive retrospective analysis was performed to elucidate the sequential stages used to establish a hospital-based EoL and bereavement program, offering a pragmatic roadmap for its implementation.

### IRB or ethical approval

The Institutional Ethics Committee of the Fundación Valle de Lili reviewed and approved the research protocol for this study (Protocol No. 1473). Our study fully adheres to the relevant Colombian regulations: Article 11 of Resolution 8430 (1993) issued by the Colombian Ministry of Health stipulates that studies presenting no risks to participants are exempt from obtaining informed consent. Our research aligns with this classification. Furthermore, Article 16, paragraph 1 of the same resolution empowers the ethics committee of the research institution to grant an exemption from obtaining informed consent when justified, specifically in the case of research devoid of risks. This study is retrospective, no clinical chart review took place and does not contain any individual personal data, therefore no patient consent to participate was needed, and no administrative permissions were required to access raw data.

### Pediatric palliative care program development

The University Hospital at Fundación Valle de Lilli (FVL) is a tertiary, nonprofit general-teaching hospital in Cali, Colombia, that provides pediatric services for children in the Southwestern region of the country. Currently, it has 668 beds, of which 35% are for pediatric patients. Highly complex services are offered, with units for pediatric oncology, bone marrow transplant, solid organ transplant, and intensive care. In 2017, the hospital established a pediatric palliative care (PPC) program called “Cuidando de Ti” (In English, “Taking Care of You”), one of the first dedicated PPC interdisciplinary teams in the country [33]. This team of two physicians, a nurse, a psychologist, and a social worker provided inpatient and outpatient patient care. It addressed physical psychosocial and emotional needs of both the patient and family during the trajectory of the disease and at the EoL. Additionally, the “Taking Care of You” (TCY) team offered basic grief and bereavement support, including follow-up calls, condolence letters, and support groups (twice per year) as needed [34]. Nevertheless, a standardized bereavement program was not yet available. Therefore, a dedicated EoL care and bereavement program was implemented in 2019, two years after launching the TCY program.

### Program implementation management: EoL care and bereavement care program development

**Fig. 1:**
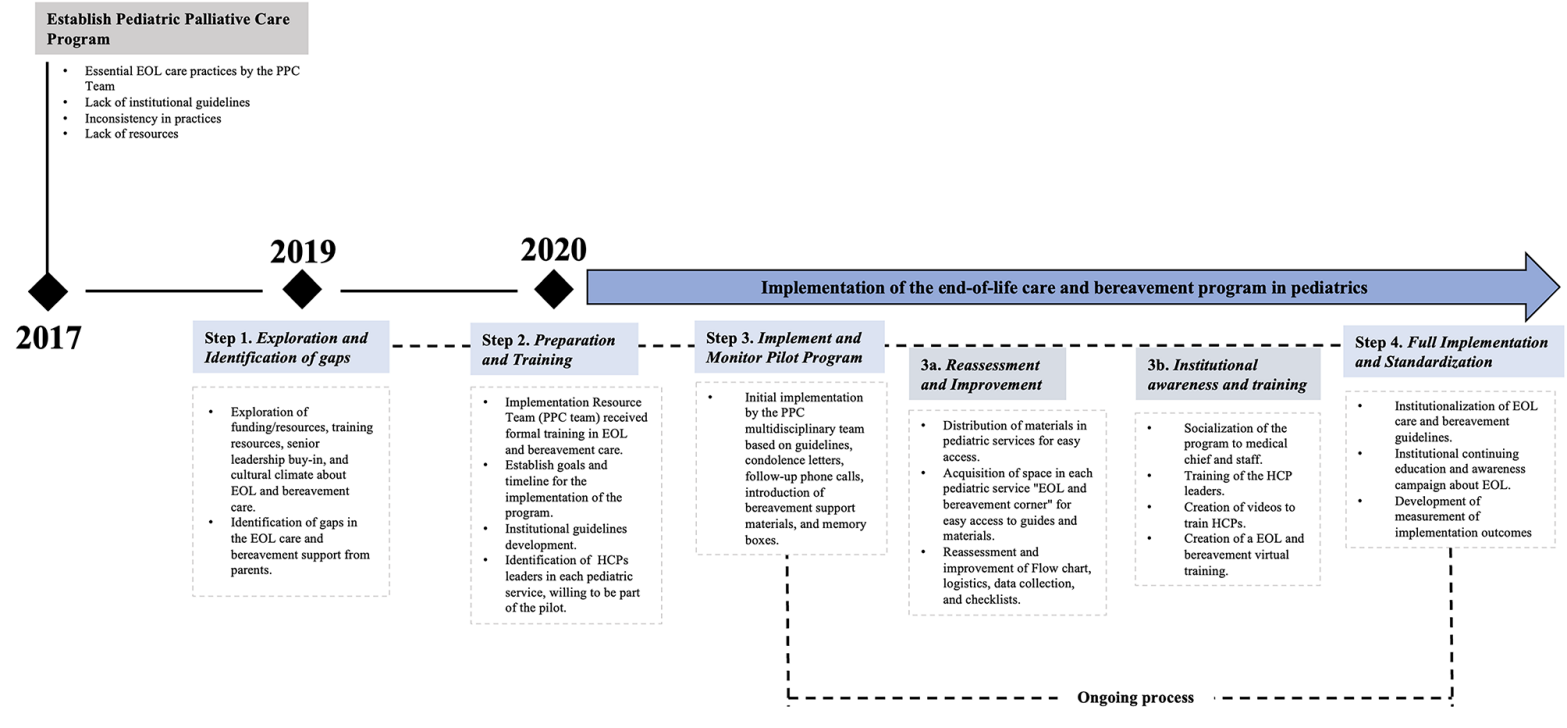
Implementation process map of the EOL Care and Bereavement Program in FVL

Program implementation is a dynamic, iterative process involving multiple overlapping steps the use of various strategies and key components [35]. Consequently, the EoL and bereavement care program implementation is described in several stages in Fig. 1. The program was designed on the premise that actions taken before and during the EoL for a patient will impact the grief process of parents and families. In other words, compassionate and quality care at the EoL may influence the trajectory of grief. Therefore, our program included interventions before and after the child’s death. As defined by Kintzel et al., EoL encompasses “the hours or days preceding imminent death, during which the patient’s physiological functions wane“ [36]; for our team, this period was approximately the last 72 h of the child’s life. The program was funded through two sources. A philanthropic donation from bereaved parents provided memory boxes and educational materials for participating families. Additionally, the My Child Matters 2019–2021 grant, supported group workshops (including multidisciplinary team participation, music therapy, materials, and a butterfly release ceremony) as well as individual psychological counseling for parents identified with complicated grief.

#### Step 1: exploration and identification of gaps

The first step involved to explore and identifying gaps. A key element that led the PPC team and the medical directors to recognize the need to improve and standardize the bereavement program was the collaboration with parents whose children had died. In November 2019, the involvement of bereaved parents became crucial as their insights, mentoring, and advice to the PPC program’s leaders at FVL. Parental feedback was collected through two primary mechanisms. First, through meetings with Colombian parents who had lost their child in an institution abroad. They shared their experiences and expressed their desire to support children and families in FVL hospital, even by providing donations of educational and memory collection materials. Second, feedback was gathered from parents whose children had died at FVL through phone-calls and bereavement workshops.

#### Step 2: preparation and training

Soon after identifying step 1, the TCY program leaders received training in EoL and bereavement care through “Resolve through Sharing” in Texas (USA). Mentoring support was also provided by other experienced hospital bereavement programs in the United States (Start Legacy Foundation, MedStar Washington Hospital Center, and St Jude Children’s Research Hospital). Various topics and tools were discussed and shared, such as hospital guidelines in EoL and bereavement, protocols, checklists of their hospitals, strategies for accompanying bereaved parents, and experiences in program standardization. Goals and timelines for the program were proposed.

Identification of champions who recognized the importance of the topic was a major challenge. Once they were identified, guidelines and management protocols were developed for each service involved: Pediatric Intensive Care Unit (PICU), Neonatal Intensive Care Unit (NICU), Emergency Room (ER), inpatient, and outpatient clinics. (Appendix A)

#### Step 3: implement and monitor pilot program

In April 2020, the pilot phase of the EoL and bereavement program began. The multidisciplinary PPC team started to apply the guidelines, send condolence letters, make follow-up phone calls, and introduce the bereavement support materials and memory boxes. The following month, internal evaluation and improvement strategies included identifying new team champions, developing training materials for FVL staff, creating flowcharts, improving logistics, creating registers and checklists.

Descriptive statistical analyses were performed on the services provided, and thematic analysis was conducted on the gratitude notes written by parents and family members who participated in the bereavement workshops [34]. This allowed the PPC team to begin self-assess its performance.

#### Step 4: full implementation and standardization

In June 2020, the dissemination and scalability of the program began being focused on training the previously identified champions in each pediatric ward. The learning process was developed through lived experiences and feedback. In subsequent months, awareness-raising for program improvement and the mandatory institutional course on EoL care and bereavement were implemented, with the development of evaluation metrics. Gradually, these practices were extended, enabling all pediatric wards at the hospital to offer comprehensive EoL care.

## Results

During the implementation period of the EoL care and bereavement program from 2019 to 2021, 606 children died in the hospital. Among these, 319 children (52,6%) had been assessed by the TCY team, and 258 (80.8%) of them were followed up by the EoL and bereavement program, since a significant portion of the hospital’s population resided in remote areas or had limited access to follow up by post mail, e-mail or even telephone calls (Table 1).

**Table 1:** Characteristics of children cared for by EOL and Bereavement Care Program (2019–2021)

Characteristics	2019–2021
	Frequency *n* = 319 (%)
**Children cared by the EoL and bereavement care program**	
< 1 year	190 (59.6)
> 1 to 5 years old	42 (13.2)
> 6 to 10 years old	37 (11.6)
> 11 to18 years old	50 (15.7)
***Bereavement for parent’s follow-up***	258 (80,8)
2019	66 (25,6)
2020	90 (34,9)
2021	99 (38,4)

* Note: Children who passed away at home or in another institution but were part of our palliative care program were also included in the bereavement support program

### Components of the EOL and bereavement program

The methodology section outlines a step-by-step approach to the development of our implementation strategy format and the components of the EoL and bereavement care program. These strategies and deliverable outcomes were classified into four domains. (Table 2).

**Table 2:** Key domains illustrating implementation strategy and deliverable outcomes of the end-of-life care and bereavement program

Domains	Definition	Implementation Strategy	Deliverable Outcome
**External Environment**	External factors of the organization can have influences on the implementation of the intervention	-Conduct educational meetings with each pediatric service of the hospital. -Promote adaptability. -Distribute educational materials. -Connect national policy about grief and bereavement program with the implementation of the program. -Involve bereaved parents’ needs and feedback in the implementation of the program.	-956 HCPs Trained in EoL and bereavement care. -10 Support workshops for bereaved parents (2–3 times per year). -150 Individual psychological follow-up for parents with risk factors for complicated grief.
**Internal Environment**	Aspects of the organization’s functioning and structure that influence the intervention’s success.	-Identification of HCPs leaders in each pediatric service who are willing to be part of the pilot. -Implementation Resource Team (PPC team) received formal training in EOL and bereavement care.	-Clinical guideline on EoL and bereavement care. -79 Personalized condolence letters. -214 Created memory boxes. -258 Follow-up calls to bereaved parents.
**Personal Attributes**	Level of knowledge, belief, attitude, and engagement of providers during implementation that may determine the intervention’s success.	-Advisory and mentoring sessions with external experts in bereavement programs. -Identification of HCPs leaders in each pediatric service who are willing to be part of the pilot. -Organizational socialization of the program to medical chief and staff.	-Inclusion of 2 trained volunteers in the end-of-life and hospital bereavement program.
**Process**	Decision making of the organization’s agents who influence the program’s implementation.	-PPC team regular meetings to evaluate and reassess the process. - Establish goals and timeline for the implementation of the program. - Institutional implementation of EoL and bereavement signaling. -Distribution of materials in pediatric services for easy access. -Acquisition of space in each pediatric service for “EOL and bereavement corner” for easy access to guides and materials.	-Developed a program implementation process map. -Created hospital database of deceased children in the institution. -Created institutional flowchart for EoL and bereavement care in pediatric services.

Adapted from:

[37] Damschroder LJ, Aron DC, Keith RE, Kirsh SR, Alexander JA, Lowery JC: Fostering implementation of health services research findings into practice: a consolidated framework for advancing implementation science. *Implementation Science* 2009, 4(1):50

EoL: End-of-Life; HCPs: Healthcare providers

#### Management and education services to healthcare providers

***Clinical guidelines and EoL care checklists*** were designed to outline the main task for supporting children and their families. The checklist and tools were distributed in each pediatric ward as a quick-reference package to allow all children in need to access the necessary care (Appendix A).

***Signaling EoL and bereavement care***. A “fallen leaf” sign was placed on the doors or windows of rooms where patients were receiving EoL care. This informed all staff that a patient and family were experiencing EoL or bereavement care, reinforcing and promoting a calm, private, respectful, and compassionate environment.

***Training and education*** were other essential components of the program. Initially, voluntary courses were organized for champions willing to support this initiative. Over time, healthcare providers (HCPs) at FVL received mandatory EoL and bereavement training. At this point, the challenge was to standardize the program, which took time and much effort.

***Databases of deceased children in the institution*** were created and updated to follow up on bereaved families and offer comprehensive support and guidance to bereaved parents and siblings.

#### Direct actions with patients and families

Six direct actions were implemented to accompany patients at EoL and families after the death of their children, as listed in Table 3.

**Table 3:** Strategies implemented to support and follow up on bereaved parents

1. Telephone follow-up
2. Identification of red flags for complicated grief
3. A personalized condolence letter sent to the home or by Email
4. Delivery of educational materials for parents on grief
5. Individualized psychological consultation for those parents with risk factors or red flags for complicated grief.
6. Group workshops for bereaved parents

***Telephone follow-up*** was an initial tool used for support. Two hospital volunteers already part of the TCY program were trained by TCY leaders in communication, grief, and bereavement care. These volunteers conducted follow-up calls, focusing on parents’ needs and offering the necessary support. A total of 258 follow-up calls were made to bereaved parents. Communication guidelines and a list of red flags for complicated grief (Appendix A) were included to determine the type of follow-up needed for each parent. Families identified as being at risk for complicated grief received individual psychological support from the PPC team psychologist.

***A personalized condolence letters and attending funerals (when possible)***, were also integral part of the EoL and bereavement care components at FVL, demonstrating ongoing support throughout the bereavement phase. In total, 79 letters of condolence and 214 memorial boxes were sent, and delivered.

***Psychological follow-up sessions*** were conducted by the team’s psychologist (up to 3 sessions). On average, the follow-up covers the first year after the child’s death, although some families require support beyond this time, depending on their needs. As a result, 150 individual psychological follow-ups were conducted for parents with complicated grief.

***Group workshops for bereaved parents.*** A total of 10 group support workshops were held. Parents were invited through follow-up telephone calls. These workshops were conducted every four months, lasted three hours each, and were led by the PPC team (PPC pediatrician, family physician, psychologist, social worker, and nurse).

During group workshops, four main activities were organized. The first activity involved sharing experiences during the trajectory of the illness and after the death of the loved one, accompanied by a psycho-education session. The second was music therapy, allowing participants to manage emotions such as anxiety, stress, and sadness. The third activity involved expressing gratitude in three areas: gratitude general, to the child, and to the health care team [34]. Finally, each family released a butterfly to commemorate and remember their child.

Parents evaluated the bereavement program positively, with comments such as “during this process, I am thankful for…. *“making us feel that we are not alone; thanks to the whole “Taking care of you” program*,* the human quality of the group”*. Families were also able to express their gratitude to their deceased child by thanking them in notes, for example, “*my daughter for the love she always showed me*,* for teaching me to fight very hard no matter the task*,* to have a big heart*,* not to complain about the little things*,* to be strong and not to decay*.”

### Awareness campaign and education of staff

Two fundamental points were essential for developing and implementing the EoL and bereavement program. First, various strategies were developed to raise awareness at all levels of care, including medical directors, staff, patients, families, and the community. These strategies included brochures, institutional ground rounds, articles in a local newspaper, and individual discussions with staff. Second, considering that PPC and bereavement care are topics with little recognition, a mandatory institutional course on EoL care and bereavement was mandated for all HCPs.

A EoL and bereavement care e-learning course was designed to provide essential knowledge to HCPs at FVL who treat patients in critical condition, with advanced illnesses, or at the EoL in an intra-hospital setting. The course content included four modules covering communication skills for delivering bad news, symptom management at the EoL, decision-making at the EoL, bereavement support, and self-care for HCPs. Acknowledging the demanding nature of caring for children at the EoL and providing bereavement support, our e-course emphasizes self-care and resilience tools to prevent burnout and compassion fatigue, ensuring the program’s sustainability. This course became mandatory for all HCPs at FVL between September 2020 and September 2022. During this period, a total of 956 HCPs were trained.

**Fig. 2:**
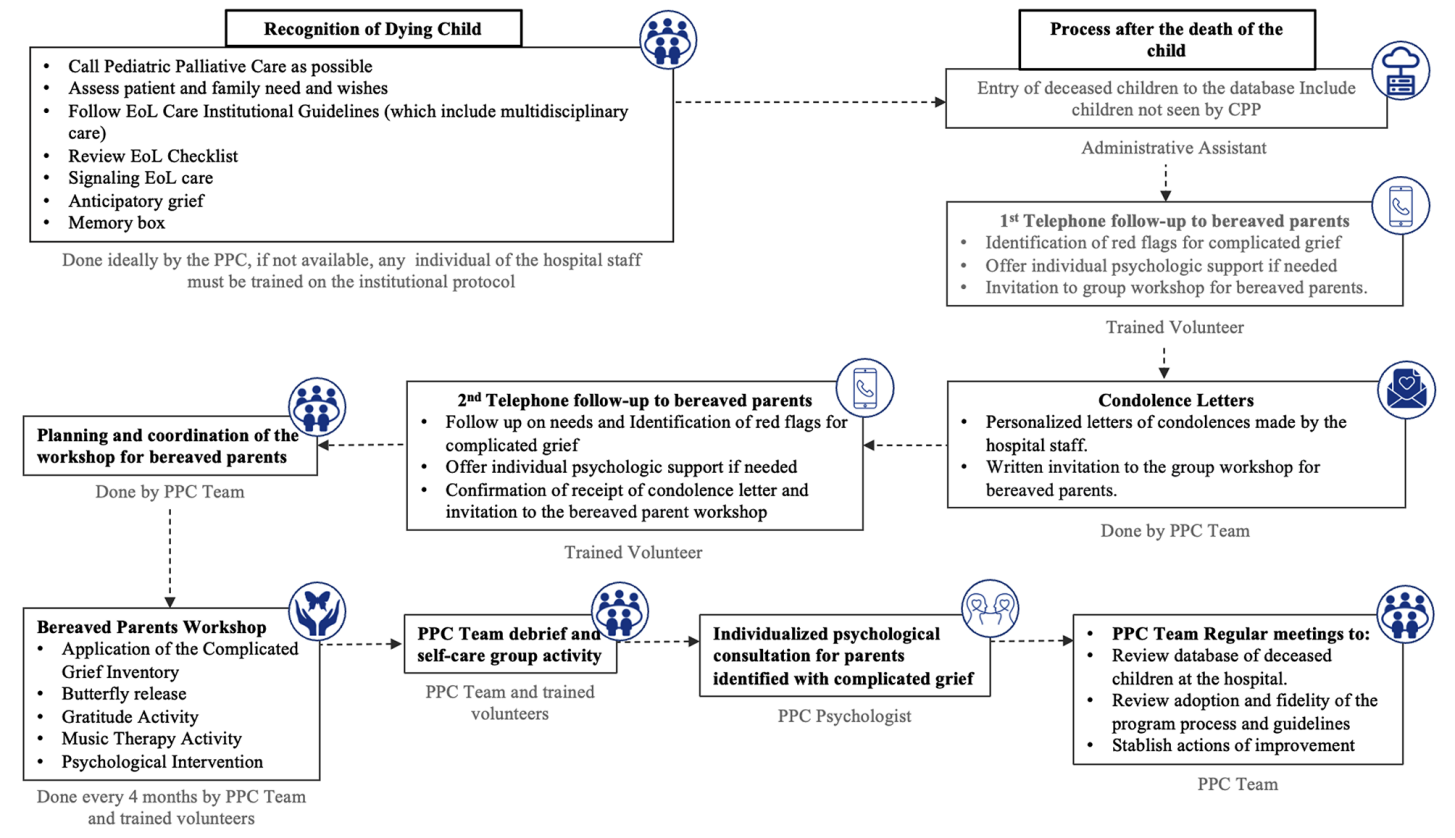
End-of-Life (EoL) and bereavement care in the pediatrics services of the hospital

Finally, one of the most used deliverables was the development of the EoL and bereavement care flowchart for pediatric services of the hospital **(**Fig. 2**)**, which facilitates understanding of the process, roles, and staff interactions.

### Adaptation of program activities during the pandemic

During the COVID-19 pandemic, some adjustments were made. Although the implementation strategies and the pilot plan continued, the TCY program introduced virtual strategies to support bereaved families. These included synchronous group calls via Zoom, delivering butterfly cocoons to parents’ homes as a symbol of life transformation, providing support material for bereaved families, and follow-up phone calls. The EoL care for children at the hospital was continued, with adjustments to ensure that the caregivers and HCPs used personal protective equipment and other measures to reduce transmission risk while applying program strategies and multidisciplinary support. Training and capacity-building courses for HCPs were maintained, primarily in a virtual setting, though one-on-one follow-ups between the PPC team and the service leaders were offered when necessary.

For bereaved parents and family members, virtual follow-up became the preferred option. Workshops for parents and the “Day of Remembrance” were held through virtual meetings that were well received by participants.

### Challenges and keys to success

While executing the pilot of our EoL and bereavement care program, several challenges were identified, such as lack of recognition and resources. Table 4 synthesizes the most significant challenges and key success factors for the program’s implementation for our team at the hospital.

**Table 4:** Summary challenges and key factors of success for the implementation of the bereaved program

Summary of Challenges	Key factors of success
Lack of recognition that EoL matters in healthcare and cultural perception of death as a taboo	- Implement awareness campaigns at all levels of care, targeting medical directors, staff, parents, and communities. - Prioritize education on the meaning of death and compassionate care throughout all stages of life, including death.
Lack of resources	- Collaborate with bereaved parents advocate and leverage philanthropic funds. - Consider grant funding, such as the “My Child Matters” grant.
Standardization of the program	- Gain support from the hospital’s medical directors. - Conduct mandatory EoL and bereavement training. - Distribute educational resources, and tools to all pediatric services. - Identify and train leaders in each ward.
Lack of time and staff	- Raise awareness among HCPs about the importance of their role in EoL and bereavement care. - Provide training in communication to facilitate compassionate care for EoL patients and their families.

FVL: Fundacion Valle del Lili Hospital. HCPs: Health care providers

## Discussion

The methods and results described in this paper suggest that it is feasible to implement a program that provides hospital-based EoL care and support for bereaved parents and families despite the multiple challenges and scarce resources in LMIC countries.

Our study identified essential components for creating and enacting a bereavement program in a hospital setting, including awareness of the gap, education, and training for HCPs across all aspects and levels of care, and collaboration with bereaved parents. The involvement of parents was essential for our program, as seen in other programs developed in HICs [20, 23, 26].

As Morris and colleagues proposed bereavement care programs as a preventative measure for bereaved families, understanding that they are at risk of developing negative outcomes, our program is also focused on supporting all families facing this difficult path, both acutely and preventively.

The primary implementation strategy of our program was to raise awareness among HCPs at FVL. Equipping HCPs with adequate EoL and grieving resources empowered them to offer compassionate support and promote their involvement in caring for these families. Additionally, early identification of risk factors for families and subsequent intervention is a critical aspect of bereavement follow-up, as highlighted by Morris et al. [20]. Therefore, promoting awareness and education about these concepts remains a fundamental component of our program.

Our program provides services and interventions to bereaved families similar to those offered by various hospitals in HICs, such as follow-up telephone calls, notes/letters of condolence, memory boxes, and bereavement workshops [18, 19, 24, 26]. Thus, this paper echoes the findings of a recent systematic review, which indicates that hospitals in LMICs might provide some form of bereavement support, despite the limited literature available [27]. Therefore, our focus is to enhance EoL care for children and their families in LMICs and to encourage sharing their work with colleagues and stakeholders globally.

The descriptive analysis of our program development enabled us to identify key factors influencing its implementation. In our study, for example, we retrospectively determined and evaluated the outlined key domains, strategies, and outcomes to organize and analyze the data.

Strong leadership and commitment serve as fundamental facilitators, securing resources and driving implementation. A straightforward program definition with measurable goals ensures a focused and successful execution, while a multidisciplinary team approach guarantees comprehensive care. Cultural competence tailors the program to diverse needs, fostering trust.

Evidence-based practices ground the program in proven interventions, enhancing care quality. Effective communication and education are crucial for buy-in and success, and supportive hospital policies facilitate program delivery. Partnerships and collaborations broaden the program’s reach. Other enablers include a positive shift in hospital culture, patient and family engagement, continuous quality improvement, and advocacy efforts. This comprehensive framework ensures a robust and adaptable implementation strategy, addressing diverse aspects crucial for the success and sustainability of our EoL care and bereavement program, potentially serving as a model for other hospitals globally.

A variety of challenges and barriers were identified by the PPC team, such as education needs for HCPs, lack of educational resources to educate on EoL and bereavement topics, absence of clinical practice guidelines, and the perceived time constraints by HCPs to implement EoL care for children and families. Additionally, a subset of families received close psychotherapeutic intervention via telemedicine during the implementation phase. However, follow-up attempts for some families were unsuccessful due to their remote location from the hospital and limited access to technology (telephone or internet). This limited the bereavement team’s ability to reach these families.

Financial constraints are a major hurdle to implementing a bereavement program. However, demonstrating that bereavement care is an essential component of care, and a public health right is key to ensuring sustainability. The commitment of healthcare providers, bereaved families, and medical directors at FVL, along with the incremental results of the program described, has led the hospital to integrate the bereavement program into its budget.

### Strengths and limitations

This work presents both strengths and limitations. To our knowledge, this study presents a novel hospital-based EoL care bereavement program established in LMICs. It offers a foundational framework of essential components adaptable to local contexts and highlights the value of stakeholder collaboration. Nevertheless, while retrospective data collection introduces potential bias, we employed mitigation strategies such as cross-referencing our databases with different institutional information sources to improve data quality. The evaluation of program sustainability is ongoing. This unique experience contributes to the global knowledge on EoL and bereavement programs.

## Conclusions

This study highlights the feasibility of developing and implementing EoL care and bereavement programs within hospitals in LMICs. Lack of resources, staff, and training are some of the identified challenges to implementation. Utilizing methodological tools allows us to identify facilitator factors and deliverable outcomes of our comprehensive EoL and bereavement program. This model provides a valuable framework for resource-limited settings.

## Future directions

This manuscript describes an EoL and bereavement program in an LMIC and lays the groundwork for a quality improvement package for EoL and bereavement care in resource-limited hospitals. Although bereavement care is already considered a standard of care for parents and families who have lost their children, it must be standardized globally, regardless of a country’s resources. Quality assessment data of our program will come in a future manuscript. We invite you to use the resources and tools this article describes to create your own programs. Our strategic initiative moving forward is to design a quality improvement package to support other LMIC hospitals in improving EoL and bereavement care adjusted to their context.

### Electronic supplementary material

Below is the link to the electronic supplementary material.

Supplementary Material 1

## Data Availability

All data generated or analyzed during this study are included within the manuscript of supplementary information files.

## Declarations

Human Ethics and Consent to Participate declarations: Not applicable.

## Abbreviations

EoL: End-of-life
ER: Emergency Room
FVL: Fundación Valle del Lili
HCPs: Health care providers
HIC: High-income country
LMIC: Low-and middle-income country
NICU: Neonatal Intensive Care Unit
PICU: Pediatric Intensive Care Unit
PPC: Pediatric palliative care
SIOP: International Society of Paediatric Oncology
TCY: Taking Care of You program
