# Supplementary material for: Implementation of a hospital-based end-of-life and bereavement care program in a latin American middle-income country. A source of light and compassion in the midst of cloudy times
Source: BMC Palliat Care. 2024 Jul 29;23:190. doi: 10.1186/s12904-024-01522-3 (PMC11285602; doi:10.1186/s12904-024-01522-3)
Supplement: Supplementary file 1 — Supplementary Material 1 [file 12904_2024_1522_MOESM1_ESM.docx]

Manuscript Title: Implementation of a hospital-based end-of-life and bereavement care program in Latin American middle-income country. A source of light and compassion in the midst of cloudy times.

**Appendix A**

**Annex 1.** Standards of care at the End of Life (EoL) in pediatric and perinatal services

**Annex 2.** End-of-Life (EoL) Checklist

**Annex 3.** Communication guide for providing End-of-Life (EoL) and bereavement care to pediatric patients and their families at the hospital

**Annex 4.** Recommendations for breast care for mothers whose child dies during the perinatal phase

**Annex 5.** Guidelines for creating lasting meaningful memories in the hospital

**Annex 6.** Condolence phrases for families whose children have died

**Annex 7.** Condolence Letter Template

**Annex 8.** Guidelines and script for a follow-up call to bereaved parents

**Annex 9.** Summary of activities conducted in group workshops for bereaved parents

**Annex 1.** Standards of Care at the End of Life in Pediatric and Perinatal Services

| **Annex 1.** Standards of care at the End of Life (EoL) in pediatric and perinatal services | |
| --- | --- |
| *1. General Recommendations* | |
| - Remember! There is always something to do! - Be caring and compassionate to the patient and family. - For the creation of memories, you must have received previous training from the Pediatric Palliative Care team. - Seek support from one of the service leaders or call the contacts below. - Allocate a necessary and thoughtful duration for creating lasting memories. Recognize that these moments often constitute the final, and sometimes sole, recollections for both families and caregivers. - When presenting any bereavement materials, kindly complete the activity registration on the attached list. | |
| *2. Contact list of the Pediatric Palliative Care team and bereavement leaders* | |
| Service Leader | Contact |
| Pediatric Palliative Care Office  Pediatric Palliative Care MD  Palliative Care Psychologist  Bereavement Nurse lead in Gynecology-Obstetrics room  Bereavement Nurse lead in NICU  Bereavement Nurse lead in General PICU  Bereavement Nurse Lead in Cardiology PICU  Bereavement Nurse lead in Pediatric Ward and Pediatric Oncology Unit |  |
| *3. Activate multidisciplinary care* | |
| - Provide spiritual support tailored to the family's beliefs and preferences (e.g., seek assistance from the institutional priest). - Ideally, seek support from the palliative care team, including a pediatrician, psychologist, nurse, and social worker. | |
| *4.* Create a supportive environment for the patient and family | |
| - Ensure that the family’s privacy is respected. - Identify the patient's end-of-life area with the *purple card* that has a “fallen leaf” (in-patient room, NICU crib, ER/PICU/cubicle door). - Allow parents to take small but meaningful actions regarding the care of the baby/child. - Uphold the privacy and individuality of the patient and their family. - Encourage and support continuous accompaniment by the child’s parents, family, and caregivers. - Minimize disruptive noises and avoid unnecessary interventions. | |
| *5. Enhance and prioritize patient comfort measures as much as possible* | |
| - Encourage physical contact of the child with parents/caregivers. - Ensure effective control of symptoms (pain, dyspnea, bleeding, emesis, etc.). - Maintain patient hygiene through gentle movements. - Keep mucous membranes adequately moist. - Regularly reassess medical interventions considered futile or distressing for the patient, in agreetment with the primary team and treating physicians (e.g., oncologists, intensivists, neonatologists, palliative care specialists). - Consider withdrawal of interventions such as chest tubes, mechanical ventilation, renal replacement therapy, nasogastric tube, and other elements that may limit the patient's comfort. | |
| *6. Use appropriate language* | |
| - Use compassionate and careful language (See communication and expressions guideline. Annex 3). | |

**Annex 2.** End-of-Life (EoL) Checklist

| **Annex 2.** EoL Checklist | | | | | | |
| --- | --- | --- | --- | --- | --- | --- |
|  | **Task** | **Y** | **N** | **N/A** | **Initials** | **Comments** |
| **Before Death** | | | | | | |
| **Prognosis Awareness** | Explain to parents the diagnosis, prognosis, possible disease trajectories, and causes of death. |  |  |  |  |  |
| **Psychology Assessment** | Emotional support |  |  |  |  |  |
|  | Anticipatory grief |  |  |  |  |  |
| **Nurse Assessment** | Ensure comprehension and reinforce key concepts. |  |  |  |  |  |
| **Social Work Evaluation** | Family support and network assessment |  |  |  |  |  |
|  | Determine the individual or family member responsible for coordinating funeral arrangements |  |  |  |  |  |
|  | Verify eligibility for funeral assistance. |  |  |  |  |  |
| **Institutional Identification of the Patient at the End of Life** | Place the purple fallen leaf sign on the door of the room. |  |  |  |  | Allow the patient and family to remain alone for a period. |
| **Provide Educational Support Materials** | Present the folder to the parent or caregiver. |  |  |  |  |  |
|  | Review and explain the content of the educational support folder |  |  |  |  |  |
| **Organ Donation** | If the parents agree, proceed to contact the relevant department. |  |  |  |  |  |
| **Memory Box** | Obtain the patient’s name. |  |  |  |  |  |
|  | Retrieve a lock of hair. |  |  |  |  | Keep it inside the memory box |
|  | Photos |  |  |  |  | Informed Consent |
|  | Capture footprints and/or handprints. |  |  |  |  |  |
| **Spiritual Support** | Ask whether the parents desire spiritual support. |  |  |  |  |  |
| **Pain and other symptoms Assessment and Management** | Perform a standardized and age-appropriate pain and other symptoms assessment scale to ensure adequate management. |  |  |  |  |  |
| **Parents’ Wishes** | Discuss with the parents whether they would like the opportunity to hold, cradle, and spend time with their baby. |  |  |  |  |  |
|  | Discuss with parents whether they would like to capture memories of the moment. |  |  |  |  |  |
| **Ensure and Respect Privacy** | Ensure that the patient is placed in an individual hospital room or cubicle. |  |  |  |  | Explain the method used (social isolation, screens, etc.) |
| **After Death** | | | | | | |
| **Informed Consent Forms** | Autopsy (if applicable) |  |  |  |  | Please provide instructions regarding the handling of the baby's remains (e.g., cremation, funeral arrangements, etc.) |
|  | Genetic studies (if applicable) |  |  |  |  |  |
| **Notifications** | Fill out the death certificate. |  |  |  |  |  |
|  | Inform the Pediatric Palliative Care Bereavement Coordinator. |  |  |  |  |  |
|  | Notify the funeral home. |  |  |  |  | Provide them with information |
| **Safety and Security** | Call when the patient is going to leave the floor to go to the morgue. |  |  |  |  | The patient must be identified with a bracelet and must be properly positioned inside the carrying bag. |
| **Upon Leaving Hospital** | Death certificate |  |  |  |  | Hand it over to the parents. |
|  | Memory box |  |  |  |  | Hand it over to the parents. |
|  | Folder with grief support information |  |  |  |  |  |
|  | Grief coordinator telephone contact |  |  |  |  |  |
|  | Inform about the ongoing follow-up that the Pediatric Palliative Care program will conduct, including follow-up calls and invitations to join support groups. |  |  |  |  |  |

**Annex 3.** Communication guide for providing End-of-Life (EoL) and bereavement care to pediatric patients and their families at the hospital

| **Annex 3.** Communication guide for providing End-of-Life (EoL) and bereavement care to pediatric patients and their families at the hospital | |
| --- | --- |
| Communication plays a vital role in providing compassionate and humanized healthcare. Here are recommended phrases for different moments of interaction with the parents of the deceased patient. | |
| Initial contact after the patient has died | *"Ms. __, my name is________, I will be your nurse/physician for the next 8 hours. I'm truly sorry that we're meeting under these circumstances, but I want to assure you that you are in good hands. Our team and I are here to provide support throughout this process."*  *"We've had the privilege of helping other parents who've gone through similar situations. If you're comfortable, would you like to share what you're thinking or feeling right now? This is your time, and we're here to offer comfort."*  *"It's completely understandable that you're feeling this way. I can sense the deep love you have for your (baby/child/son/daughter) and the strong bond you've created. The news is incredibly shocking and goes beyond our or your imagination."* |
| Praise/encourage the parent/caregiver, when possible | *"I can see the deep care and love you've given your child [mention their name if known]."*  *"You made a thoughtful decision to come to the hospital due to [pain, fever, and/or bleeding, etc.]."*  *"You've shown immense love and care as parents. [Name of the patient] was fortunate to have you by their side."* |
| If the parent/caregiver is alone, seek for support | *"Is anyone aware of your child's admission to the hospital?"*  *Or, "Do you have someone you'd like me to contact for you?"* |
| Introduction and delivery of bereavement materials and memory box | *"Ms._________, I cannot imagine what you are going through. Our hospital is committed to supporting you in this difficult time by offering all the resources available to make this situation as bearable as possible. We have provided some reading materials that may assist you in coping with the loss of your child. Furthermore, we've prepared a special keepsake box in honor of your child. Inside, you'll find meaningful mementos. Take your time going through the box, and feel free to add items whenever you feel ready. You can also take the box home with you and continue adding items if you wish."* |
| Encourage and allow parents to spend time with their baby/child | *"We understand the importance of parents spending time with their baby to build a connection and create additional memories. Please be aware that you have unrestricted time with your baby."* |
| Offer psychological and/or spiritual support | *"We offer emotional and psychological support services, as well as spiritual care if desired. If you would like, we can arrange a visit with our team psychologist and/or the hospital chaplain for prayer or additional support."* |
| Expressions that are frequently used but are **not appropriate** | 1. **"Your child is in a better place":** This is not appropriate because parents want their children by their side physically. Therefore, these words may sound cold, as the best place for children is next to their parents. 2. **"It is the will of God":** The figure of God may not be part of the beliefs or spiritual traditions of the person. Additionally, we cannot certify that it was "the will of God," which can hinder the situation's acceptance or spiritual understanding. 3. **"Time will heal":** The grieving parent may feel or think that the pain will not go away or heal. The pain does not really diminish, but it does change. Each person has their own time. 4. **"You can have another son/daughter":** Children are not replaceable, and we do not know the fertility status or desires of the couple in the face of future pregnancies. 5. **"I'm so sorry":** This language can be confusing because they may think you didn't do something for which forgiveness is necessary. In addition, it incites feelings of sadness for the loss. It is not recommended as an introduction at the time of delivering bad news, but it can be a way to comfort over time. 6. **"At least":** This term is usually incorporated into responses to news of death or loss, e.g., "at least he/she didn't suffer." This language minimizes the pain of loss.   In general:   - Avoid judging the patient or their family member. - Avoid giving advice. - Avoid comparing losses. |

**Annex 4.** Recommendations for breast care for mothers whose child dies during the perinatal phase

| **Annex 4.** Recommendations for breast care for mothers whose child dies during the perinatal phase |
| --- |
| Note: After delivering a baby over 17 weeks' gestation, most mothers will naturally produce breast milk, leading to physical and emotional challenges in the absence of a baby to breastfeed. This may result in mild to severe swelling within two to five days post delivery. |
| To alleviate discomfort from breast swelling, consider the following options:   - **Breast Ice Packs:** Alternate ice packs every 20 minutes to reduce inflammation and discomfort. - **Medications:** Consult with your doctor about taking acetaminophen or ibuprofen as directed to manage inflammation and pain. - **Tight Bra:** Wear a well-fitting bra (e.g., sports bra) day and night from delivery to minimize the discomfort of heavy breasts. - **Hot Showers:** Use heat to encourage a small amount of milk leakage for relief. Soaking breasts in warm water can also be beneficial. Use hot or warm water only if milk expression is necessary, as it may stimulate further milk production. |
| If discomfort persists after trying the above methods, manual milk extraction may be considered. Gently press on the edge of the areola, pushing back towards the chest wall, while simultaneously pressing thumb and fingers together. Repeat until the pressure is relieved. Extract only enough milk to ease discomfort, as fully emptying the breasts can stimulate increased milk production. |
| Methods **no longer recommended** include:   - **Breast Squeezing** - **Taking Medicine to "Dry" Milk** - **Limiting Fluid Intake** |
| Gradually, breasts will return to their previous size. It's normal to observe a drop or two of milk, even several months later, as these changes are a natural hormonal response. Feeling sadness when seeing milk without a baby to feed is also normal. Seek support from friends, family, and spiritual counselors to share your feelings about this experience. |

**Annex 5.** Guidelines for creating lasting meaningful memories in the hospital

| **Annex 5.** Guidelines for creating lasting meaningful memories in the hospital |
| --- |
| *Memory-making ideas:*  Ensure active parental involvement in creating cherished memories. Parents should not be observers.   - Guide parents in bathing, cleaning, and dressing their child. - Collect hand/footprints for the memory box. - Customize the memory box based on parental preferences:   - Hand/footprints   - Lock of hair   - Announcement card with completed details   - Signed sympathy card – Gather signatures from staff/management.   - Printed photos of the baby and the parents |
| *Photography guidelines for meaningful memories:*   - Complete the **informed consent** process for capturing photographs. - Allocate sufficient time for the photography session, recognizing that these pictures may be the last or only tangible memories for parents. - For newborns, take multiple photos in different positions: from 10 shots, only one may be suitable for providing to parents. - Opt for black and white photos to minimize the visual impact of physical changes post-mortem. - Favor natural shots, capturing parents interacting organically with the baby/child. - In cases of malformations or evident physical changes, prioritize capturing images of hands and feet (before taking fingerprints). - If printing is not immediately feasible, inquire about the parents' preferred contact method for sending digital copies. Allow parents to choose the most meaningful photos. |

**Annex 6.** Condolence phrases for families whose children have died

| **Annex 6.** Condolence phrases for families whose children have died |
| --- |
| Below you will find some examples of condolences phrases:   - "Even though words may not provide solace, please know that we are here for you." - "Words can never fully express the depth of our sorrow, but our presence and support are unwavering." - "May our condolences offer you some comfort, and may our prayers alleviate the weight of your grief." - "Our thoughts and well wishes are with you during this time of sorrow." - "The memory of [Child's Name] will forever dwell in our hearts." - "You and your family are in our prayers. We extend our heartfelt condolences for your loss." - "It's challenging to accept the absence of [Child's Name], but what we cherish in our hearts and memories will endure." - "Our wishes for healing and peace go out to you. Please accept our deepest condolences." - "The ones we love never truly leave us; they walk alongside us every day." - "During this difficult time, we offer our sincere condolences and stand in solidarity with you." - "The pain of this unforeseen tragedy is shared, and we will always hold the memory of [Child's Name] close." - "I'm at a loss for words, but I share in your profound grief. Accept my deepest condolences." - "I'm speechless, but I feel your pain deeply. Receive my condolences". - "Our heartfelt condolences. May God grant your family the strength needed during this challenging time." (based on the spiritual and religious beliefs or preferences of the family) - "May God envelop your family in peace and comfort throughout this difficult period. Our deepest sympathies." (based on the spiritual and religious beliefs or preferences of the family) |

**Annex 7.** Condolence letter template

| **Annex 7.** Condolence letter template |
| --- |
| Dear [Child's Name] Family,  A few weeks ago, we bid farewell to [Child's Name], and today, from the memories we shared, the entire team of the [Name of the Program] Pediatric Palliative Care program extends our deepest condolences for your loss.  We want to express our profound admiration for the dedication with which you cared for [Child's Name]. Through your commitment and love, you provided a nurturing environment for (him/her), ensuring (he/she) felt surrounded by care and affection.  We appreciate the trust you placed in our team, collaborating with us during the time we shared in caring for [Child's Name], even during the most challenging moments of pain and uncertainty.  Our team remains by your family’s side. In consideration of your well-being, we've prepared this Grief Kit, which includes emotional support materials and a butterfly—a symbol of life's transformation.  For further support, please don't hesitate to reach out to us at [Location] or call us at [Phone number].  Sending you warm hugs from each member of our team.  With affection and appreciation,  [Signatures of the PPC Team] |

**Annex 8.** Guidelines and script for a follow-up call to bereaved parents

| **Annex 8.** Guidelines and script for a follow-up call to bereaved parents |
| --- |
| **When?** (Recommended)   1. One week after the baby/patient's death 2. One month after the death 3. Four months after the death 4. Ten months after the death 5. Anniversary of the death   REMEMBER: Fill out the database for care coordination. |
| **Telephone follow-up guide:** |
| **1. Identify yourself:**   - “Hello, my name is [Name of the person who is calling], I am the coordinator of the pediatric palliative care bereavement program at [Hospital Name]”. - Mention if you met them at any time during the child's illness: “We met/I was with [Patient's name] at [Recall the time you meet that person]”. |
| **2. Mention the purpose of the call:**   - “I am calling to see how you have been since the death of [Patient's Name]. |
| 1. **Make sure is a good time for the conversation:**  - Is now a good time to talk?”. |
| 1. **Ask for general information on how they've been:**  - “Tell me how you have been coping since [Patient’s name] passed away?” “Who has been supporting you during these difficult times?” or “Have you had supportive company during this challenging period?” - Ask about the well-being of close individuals’ spouse or father of the patient has been. “How's your [spouse/father of the baby/grandparent/aunt/sibling] holding up?” - Share details about the funeral: “Did you do something special to honor [Child’s Name]?” |
| 1. **Identify red flags for complicated grief by asking more specific information:**  - Have you been able to do what you need to do in the day? [Checking safety] - Have you been able to sleep well lately? [Checking wellbeing] - How have you been eating? [Checking wellbeing] - Have you been able to return to your activities? Work? Etc.? |
| **5. Respond to shared information: If the parents share a difficult/challenging situation related to the grief period, you have a few options:**   - “What has helped you handle these situations in the past? Or “What has helped you cope with these situations?” - “Do you know who you can turn to when you feel this way?” - "It's a painful reality." - "You need time to heal." - "Time will gradually decrease the intensity of the pain."  1. **Foster support networks:**  - "The fact that your coworkers are calling you is because they care about you and want to help you" |
| 1. **Offer to help:**  - “Is there anything you believe we can help you with?” |
| 1. **Mention the risk factors identified:**  - "I am concerned that [Red Flag identified, Ex: “You haven’t been able to go back work"] |
| 1. **Close the conversation:**  - "Thank you for sharing this information with me..." - "We've had a good conversation today." - “Would you like me to call you again to check on how you are doing?” - “What is the best time for you/to call you? |
| 1. **Follow-up with Condolence Letter and invite to the support group:**   To send condolence letter home: "The [Name of the Program] team wants to honor the life of [Child’s Name] and other children we have accompanied. For this, we want to send you a specially prepared message. Could you please share your mailing address with us? We also want to send a small tribute to honor the life of [Child’s Name]. Can you confirm your address?"  Invitation to the support group: "Our team [Name of the Program], wants to honor the life of [Name of the Child] and the other children we have accompanied, for this we want to invite you to the "Release of Butterflies Ceremony" to be held on [Date] at [Place] at [Time]. We would love for you to join us! You will receive an invitation; you can confirm your address...." |
| **Risk factors to identify in the call:**   - Denial of the child's death. - General sense of fatality, inability to enjoy life: Inability to return to work, persistent isolation from friends and family, verbalization of hopelessness, or a sense of permanent emptiness, all relative to the time between the death of the child and the follow-up call. - Hostility and anger towards family or healthcare professionals/institution that cared for the baby. - Continuous rejection of help and support from others. - Prolonged feelings of guilt about the death, either their own or reflected in family members or healthcare professionals. - Self-destructive behaviors, substance abuse, thoughts of death or suicide. |
| **Expressions to avoid:**  "Your child is in a better place."  "It is the will of God."  “Don’t worry.”  “I know exactly how you feel.”  "At least..." “At least you have more children."  "At least you are young and can have more children."  "At least you didn't have to see him die," etc. |

**Annex 9.** Summary of activities conducted in group workshops for bereaved parents

| **Annex 9.** Summary of activities conducted in group workshops for bereaved parents |
| --- |
| 1. Debrief and share experiences and feelings Related to the trajectory of the child's disease 2. Educational session on grief led by the psychologist of the PPC team 3. Activity to promote and cultivate gratitude regarding the painful experience lived 4. Butterfly release activity as a symbol of life transformation. |
